# Clinical profile of patients with acute traumatic brain injury undergoing cranial surgery in the United States: report from the 18-centre TRACK-TBI cohort study

**DOI:** 10.1016/j.lana.2024.100915

**Published:** 2024-10-17

**Authors:** John K. Yue, John H. Kanter, Jason K. Barber, Michael C. Huang, Thomas A. van Essen, Mahmoud M. Elguindy, Brandon Foreman, Frederick K. Korley, Patrick J. Belton, Dana Pisică, Young M. Lee, Ryan S. Kitagawa, Mary J. Vassar, Xiaoying Sun, Gabriela G. Satris, Justin C. Wong, Adam R. Ferguson, J. Russell Huie, Kevin K.W. Wang, Hansen Deng, Vincent Y. Wang, Yelena G. Bodien, Sabrina R. Taylor, Debbie Y. Madhok, Michael A. McCrea, Laura B. Ngwenya, Anthony M. DiGiorgio, Phiroz E. Tarapore, Murray B. Stein, Ava M. Puccio, Joseph T. Giacino, Ramon Diaz-Arrastia, Hester F. Lingsma, Pratik Mukherjee, Esther L. Yuh, Claudia S. Robertson, David K. Menon, Andrew I.R. Maas, Amy J. Markowitz, Sonia Jain, David O. Okonkwo, Nancy R. Temkin, Geoffrey T. Manley, the TRACK-TBI Investigators, Jason E. Chung, Bukre Coskun, Shawn R. Eagle, Leila L. Etemad, Brian Fabian, Feeser V. Ramana, Shankar Gopinath, Christine J. Gotthardt, Ramesh Grandhi, Sabah Hamidi, Ruchira M. Jha, Christopher Madden, Randall Merchant, Lindsay D. Nelson, Richard B. Rodgers, Andrea L.C. Schneider, David M. Schnyer, Abel Torres-Espin, Joye X. Tracey, Alex B. Valadka, Ross D. Zafonte

**Affiliations:** a Department of Neurological Surgery, University of California, San Francisco, San Francisco, CA, United States; b Brain and Spinal Injury Center, Zuckerberg San Francisco General Hospital, San Francisco, CA, United States; c Departments of Neurological Surgery and Biostatistics, University of Washington, Seattle, WA, United States; d University Neurosurgical Center Holland, Leiden University Medical Center, Haaglanden Medical Center, HAGA, Leiden, The Hague, the Netherlands; e Department of Surgery, Division of Neurosurgery, QEII Health Sciences Centre and Dalhousie University, Halifax, Nova Scotia, Canada; f Department of Neurology, University of Cincinnati, Cincinnati, OH, United States; g Department of Emergency Medicine, University of Michigan, Ann Arbor, MI, United States; h Department of Neurological Surgery, University of Wisconsin-Madison, Madison, WI, United States; i Center for Medical Decision Making, Department of Epidemiology and Public Health, Erasmus MC, University Center Rotterdam, Rotterdam, the Netherlands; j Department of Neurosurgery, Erasmus MC - University Medical Center Rotterdam, Rotterdam, the Netherlands; k Department of Neurological Surgery, The University of Texas Health Science Center at Houston, Houston, TX, United States; l Biostatistics Research Center, Herbert Wertheim School of Public Health and Longevity Science, University of California San Diego, La Jolla, CA, United States; m Department of Neurobiology, Morehouse School of Medicine, Atlanta, GA, United States; n Department of Neurological Surgery, University of Pittsburgh Medical Center, Pittsburgh, PA, United States; o Department of Neurological Surgery, University of Texas at Austin, Austin, TX, United States; p Department of Neurology, Harvard Medical School, Boston, MA, United States; q Departments of Emergency Medicine and Neurology, University of California, San Francisco, San Francisco, CA, United States; r Department of Neurological Surgery, Medical College of Wisconsin, Milwaukee, WI, United States; s Department of Neurological Surgery, University of Cincinnati, Cincinnati, OH, United States; t Department of Psychiatry, University of California, San Diego, La Jolla, CA, United States; u Department of Rehabilitation Medicine, Spaulding Rehabilitation Center, Boston, MA, United States; v Department of Neurology, University of Pennsylvania, Philadelphia, PA, United States; w Department of Radiology and Biomedical Imaging, University of California, San Francisco, San Francisco, CA, United States; x Department of Neurological Surgery, Baylor College of Medicine, Houston, TX, United States; y Division of Anaesthesia, Department of Medicine, University of Cambridge, Cambridge, United Kingdom; z Department of Neurological Surgery, Antwerp University Hospital, Edegem, Belgium; aa Department of Translational Neuroscience, University of Antwerp, Antwerp, Belgium

**Keywords:** Decompressive craniectomy, Craniotomy, Glasgow outcome scale, Medical decisionmaking, Neuroimaging, Triage, Traumatic brain injury, Traumatic intracranial hemorrhage

## Abstract

**Background:**

Contemporary surgical practices for traumatic brain injury (TBI) remain unclear. We describe the clinical profile of an 18-centre US TBI cohort with cranial surgery.

**Methods:**

The prospective, observational Transforming Research and Clinical Knowledge in Traumatic Brain Injury Study (2014–2018; ClinicalTrials.gov #NCT02119182) enrolled subjects who presented to trauma centre and received head computed tomography within 24-h (h) post-TBI. We performed a secondary data analysis in subjects aged ≥17-years with hospitalisation. Clinical characteristics, surgery type/timing, hospital and six-month outcomes were reported.

**Findings:**

Of 2032 subjects (age: mean = 41.4-years, range = 17–89-years; male = 71% female = 29%), 260 underwent cranial surgery, comprising 65% decompressive craniectomy, 23% craniotomy, 12% other surgery. Subjects with surgery (vs. without surgery) presented with worse neurological injury (median Glasgow Coma Scale = 6 vs. 15; midline shift ≥5 mm: 48% vs. 2%; cisternal effacement: 61% vs. 4%; p < 0.0001). Median time-to-craniectomy/craniotomy was 1.8 h (interquartile range = 1.1–5.0 h), and 67% underwent intracranial pressure monitoring. Seventy-three percent of subjects with decompressive craniectomy and 58% of subjects with craniotomy had ≥3 intracranial lesion types. Decompressive craniectomy (vs. craniotomy) was associated with intracranial injury severity (median Rotterdam Score = 4 vs. 3, p < 0.0001), intensive care length of stay (median = 13 vs. 4-days, p = 0.0002), and six-month unfavourable outcome (62% vs. 30%; p = 0.0001). Earlier time-to-craniectomy was associated with intracranial injury severity.

**Interpretation:**

In a large representative cohort of patients hospitalised with TBI, surgical decision-making and time-to-surgery aligned with intracranial injury severity. Multifocal TBIs predominated in patients with cranial surgery. These findings summarise current TBI surgical practice across US trauma centres and provide the foundation for analyses in targeted subpopulations.

**Funding:**

National Institute of Neurological Disorders and Stroke; US 10.13039/100000005Department of Defense; Neurosurgery Research and Education Foundation.

**Research in context**

**Evidence before this study**

Clinical care guidelines for traumatic brain injury (TBI) vary across centres, regions, and countries. Emergent surgical decompression is often indicated in patients with TBI with pathological progression of intracranial mass effect. In 2020, the Brain Trauma Foundation incorporated evidence regarding the utility of surgical decompression for refractory intracranial hypertension from two large randomised controlled trials (RESCUEicp performed by the CENTER-TBI Study, and DECRA performed by the OzENTER-TBI Study) conducted primarily outside of the United States (US), which may not reflect the contemporary surgical practice for TBI within the US.

We conducted a broad search for published reports between September 1, 2004 and September 28, 2024 across the biomedical databases PubMed, Cochrane Library, Google Scholar, ScienceDirect, and Web of Science. Our search strategy comprised the following key terms: “traumatic brain injury”, AND “cranial surgery”, “craniotomy” OR “craniectomy” OR “surgical practice”, AND “United States” OR “US”. Our search yielded one retrospective study examining craniotomy vs. craniectomy in acute subdural haematomas and hospital mortality using a US national database. There were no prospective studies that comprehensively characterised the clinical risk factors, intracranial injury subtypes, surgery type and timing, and related outcomes of patients with TBI who underwent cranial surgery across representative US centres over the past two decades. This undercharacterisation contributes to the critical knowledge gap impeding the refinement of clinical best practices and targeted treatment strategies for hospitalised TBI cohorts within the US specifically.

**Added value of this study**

This secondary data analysis of the TRACK-TBI Study is the first study to rigorously and comprehensively characterise a prospectively enrolled cohort of hospitalised patients with acute TBI who underwent cranial surgery across 18 US Level I trauma centres between 2014 and 2019. Our study aimed to provide an overview of the cranial surgery cohort, inclusive of presentation, clinical and radiological injury characteristics, surgery type and timing, hospital interventions and outcomes, and six-month outcomes. Enrolment, data collection, and data coding processes and procedures in the TRACK-TBI Study were centrally standardised, and all clinical protocols are publicly available to facilitate study transparency, rigor, and reproducibility. The proportion of patients who underwent acute cranial surgery was commensurate with recent large international TBI studies including CENTER-TBI and OzENTER-TBI. Our study found strong associations between clinical and radiologic TBI severity, need for cranial surgery, and earlier time to surgery. Worse clinical and radiologic TBI severity was associated with earlier time-to-surgery specifically in patients with decompressive craniectomy. Compared to craniotomy, decompressive craniectomy was associated with longer hospital length of stay and six-month unfavourable outcome. These results descriptively summarise the current surgical practice for TBI across US Level I trauma centres and provide support for future hypothesis-driven investigations on risk stratification, prognostication, and comparative effectiveness.

**Implications of all the available evidence**

Refining TBI prognostic models with contemporary data is requisite to advance management of TBI across care settings. Our results improve the modern evidence base for future studies examining comparative effectiveness between decompressive craniectomy vs. craniotomy across traumatic intracranial lesion types, severities, and volumes, which to date has only been assessed for acute subdural hematomas. Our findings serve as the analytic foundation for deep multivariable assessment of factors that may influence surgical timing/type and outcomes, including relevant and increasingly validated predictors such as blood-based biomarkers in TBI, volumetric neuroimaging findings, frailty, polytrauma, and bio-psycho-socio-ecological variables; these factors have been shown to influence TBI prognosis, however their impact on surgical decision-making and management remains to be clarified. These studies may pave the way for improved surgical decision-making algorithms and prognostic models for the evolving casemix of patients with TBI in the US.

## Introduction

Traumatic brain injury (TBI) is a major cause of injury-related deaths worldwide, with annual incidence of 50–60 million cases and annual costs exceeding $400 billion United States (US) dollars.^1^ TBI care has been recognised as inconsistent across countries, regions, and centres, with heterogeneous contributing factors.^1^ While guidelines for TBI surgical management were provided by the Brain Trauma Foundation in 2006,^2^ modern surgical practices require updated characterisation, as demographics, clinical severity, diagnostic modalities, access to treatment, and medical information flow have changed over time.^2^

Multidisciplinary and multimodality neurotrauma care has evolved over the past two decades. The Brain Trauma Foundation Guidelines for the Management of Severe Traumatic Brain Injury Fourth Edition was updated in 2020 to incorporate findings from two randomised controlled trials: Decompressive Craniectomy in Patients with Severe Traumatic Brain Injury,^3^ and Trial of Decompressive Craniectomy for Traumatic Intracranial Hypertension (RESCUEicp).^4^ The Brain Trauma Foundation Guidelines provide best practices for management of intracranial pressure elevations. Interval updates were encouraged to establish “living guidelines” and ensure that adopted clinical practices remain relevant to their intended patient cohorts,^5^ which requires feedback from representative clinical studies.

Prognostic modelling can provide insight into efficacy and quality of guidelines-based treatment. Two extensively validated prognostic models for mortality and unfavourable outcome were developed from the International Mission on Prognosis and Analysis of Clinical Trials in Traumatic Brain Injury (1984–1997)^6^ and the Corticosteroid Randomisation After Significant Head Injury (1999–2004)^7^ studies. Recent evidence showed that these models may overestimate mortality and unfavourable outcome when applied to two large TBI studies conducted between 2014 and 2018 across 18 US hospitals (Transforming Research and Clinical Knowledge in Traumatic Brain Injury [TRACK-TBI])^8^ and 77 centres in Europe and Israel (Collaborative European NeuroTrauma Effectiveness Research in Traumatic Brain Injury [CENTER-TBI]).^1^ These findings highlight the need to refine TBI prognostic models with updated understanding of TBI casemix and surgical practices.

Recent data showed that subsets of severely-injured patients with TBI improve over years post-injury,^9^^,^^10^ prompting increased interest in surgical decision-making and surgical predictors of long-term outcome.^11^^,^^12^ The RESCUEicp trial reported that surgery for refractory intracranial pressure elevations were associated with reduced mortality at two years post-injury.^13^ Similarly, CENTER-TBI found the association between early cranial surgery and better six-month outcome for moderate TBI with intracerebral haematomas.^14^ The complex relationships between socio-demographics, medical history, intracranial injury burden, clinical decision-making, and outcomes in contemporary patients in the US require examination of rigorously-collected data from representative TBI populations.

Herein we describe the clinical profiles and outcomes of patients hospitalised with acute TBI from the 18-centre US TRACK-TBI cohort who underwent cranial surgery. We aimed to provide an overview of this cohort, including presentation, injury characteristics, surgery type/timing, acute clinical management, and six-month outcomes. Our report provides evidence for upcoming hypothesis-driven analyses utilising the surgical TBI cohort, including risk stratification, prognostication, and comparative effectiveness research.

## Methods

### Study overview

The prospective, observational TRACK-TBI Study (ClinicalTrials.gov #NCT02119182) enrolled subjects through convenience sampling across 18 US Level I trauma centres between February 26, 2014 and July 30, 2018, with final follow-up on June 30, 2019. Inclusion criteria were presentation to the emergency department with acute non-penetrating TBI meeting at minimum the American Congress of Rehabilitation Medicine definition for TBI,^15^ and clinical triage to head computed tomography (CT) scan within 24-h (h) of injury. Exclusion criteria were pregnancy, incarceration, penetrating TBI, non-survivable physical trauma as determined by the principal investigator at each study site, and pre-existing medical or neuropsychiatric conditions that could interfere with outcome assessments as determined by the principal investigator at each site. The institutional review board (IRB) at each study site approved all TRACK-TBI Study protocols, which were inclusive of secondary data analyses using TRACK-TBI Study data. Subjects or their legally authorised representatives (LAR) provided written informed consent. The Galveston Orientation and Amnesia Test was administered to each subject for competency screening to determine capacity for consent. For subjects without passing scores, LAR provided initial consent. Competency screening was repeated at each follow-up visit, and if a passing score was achieved, the subject was re-consented for participation. Secondary data analyses using TRACK-TBI Study data did not require separate IRB/ethical approval or additional consent procedures.

Data collection conformed to the National Institute of Neurological Disorders and Stroke (NINDS) TBI Common Data Elements version two.^16^ Socio-demographic and medical history, clinical, and acute hospitalisation variables were collected.^17^ Subsets of subjects underwent venepuncture for blood biomarker analyses at day one, three, five, two-weeks, and six-months, brain magnetic resonance imaging (MRI) at two-weeks and six-months, and outcomes assessment at two-weeks, three-months, six-months, and 12-months. Detailed study protocols, consent forms, and data collection forms are available on the TRACK-TBI website.^18^

The current study is a secondary descriptive analysis of subjects hospitalised with TBI from the TRACK-TBI Study.

### Cohort determination and analytic plan

Data from hospitalised subjects aged ≥17 years at time of injury were extracted from the TRACK-TBI Study. Per our aims, cranial surgery for acute TBI was our primary variable of interest. The first (i.e. index) cranial surgery included decompressive craniectomy or craniotomy to relieve mass effect, and other surgeries including skull fracture repair, skull base repair, intracranial pressure monitor placement in the operating room, debridement, or another surgery not for decompression/evacuation of mass effect. Surgical indications and criteria were not set by the TRACK-TBI Study and were determined locally at each centre, which generally conformed to the Brain Trauma Foundation Guidelines. Decompressive craniectomies were performed for supratentorial traumatic injuries in our cohort. The Elixhauser Comorbidity Index was calculated using International Classification of Diseases diagnosis codes. The non-head/neck Injury Severity Score was calculated from the sum of squares of the highest three scores amongst five non-head/neck body systems (face, chest/thorax, abdomen, extremities/pelvic girdle, external) to measure extracranial injury severity. Socio-demographic, clinical, and injury-related variables were compared between subjects with and without cranial surgery.

**Fig. 1:**
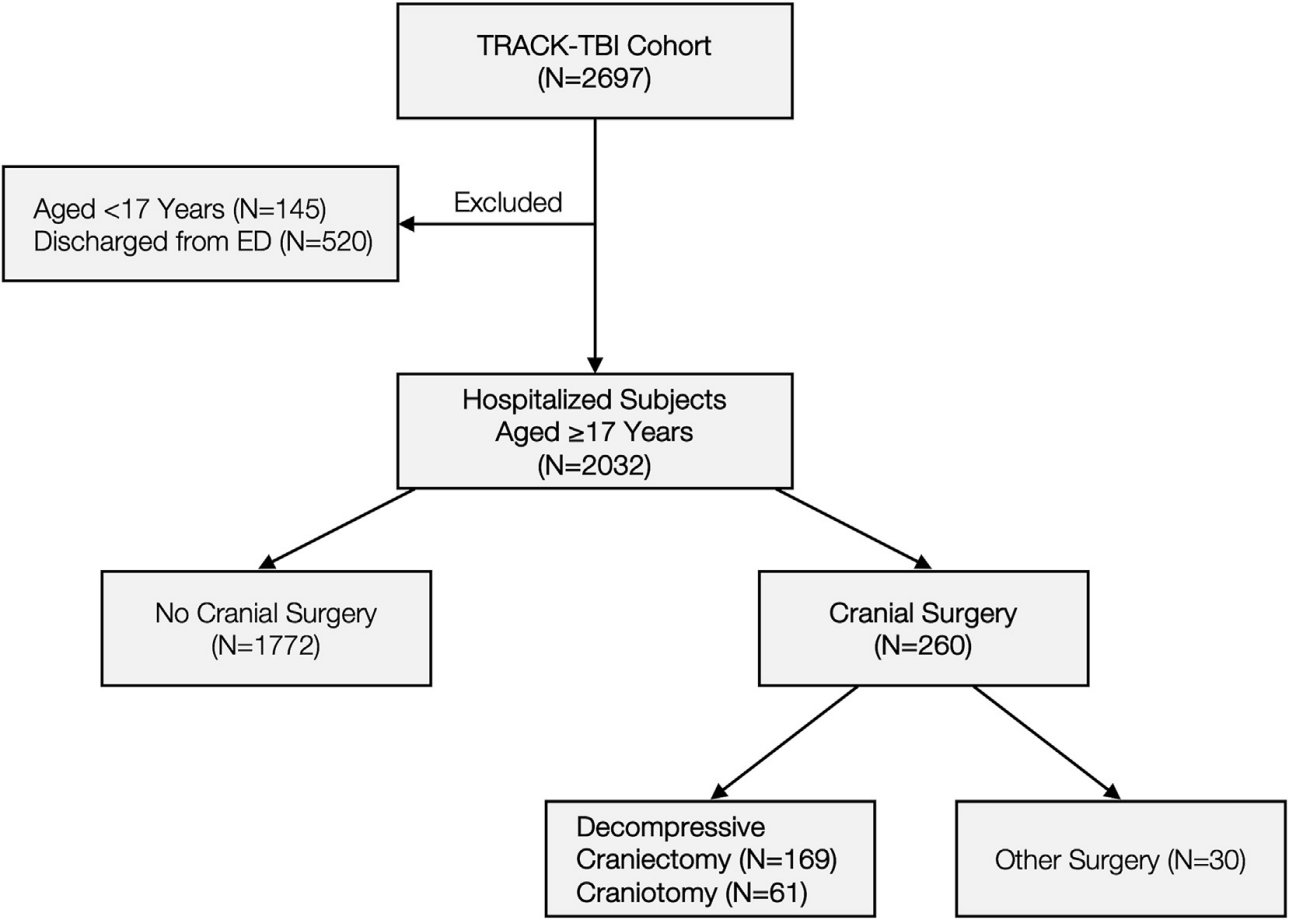
**Flow diagram of included subjects**. The CONsolidated Standards of Reporting Trials (CONSORT) flow diagram for the current study, which aimed to characterise subjects hospitalised with acute TBI who underwent cranial surgery. TRACK-TBI, Transforming Research and Clinical Knowledge in Traumatic Brain Injury.

We then focused our analysis on subjects with the primary surgical indication in TBI (decompression or evacuation of mass effect; decompressive craniectomy/craniotomy). Time-to-surgery was calculated from the time of injury. Second craniectomy/craniotomies (after the index surgery) were identified. Intracranial pressure monitor placement was coded as prior to or during/after index surgery. Hospital length of stay and intensive care unit (ICU) length of stay were reported for subjects alive at hospital discharge. For surgery timing, we examined clinical factors and outcomes by 0–2, 2–4, 4–24, and >24 h groups, in consideration of decreased mortality associated with surgery for acute subdural haematoma within 4 h discussed in the 2006 Brain Trauma Foundation Guidelines.^2^^,^^19^ The CONsolidated Standards Of Reporting Trials flow diagram is shown in Fig. 1.

### Neuroimaging data

Head CT images were de-identified at each TRACK-TBI centre, uploaded to a central repository (Laboratory of Neuro Imaging, Los Angeles, California, US), and coded in accordance with NINDS TBI Neuroimaging Common Data Elements by a central board-certified neuroradiologist blinded to clinical information except age and sex.^20^ Given its prognostic utility, the Rotterdam CT Score for structural TBI severity^21^ was coded by the same central neuroradiologist.

### Outcome measures and coding

The Glasgow Outcome Scale-Extended (GOS-E) is an overall measure of functional disability based on consciousness, independence inside/outside the home, employability, social/community participation, and post-concussion symptomatology.^9^^,^^22^ GOS-Es were administered through structured interviews by trained personnel at each outcomes timepoint to capture TBI-related disability (e.g. excluding disability attributable to extracranial injuries). The 8-point ordinal scale consists of 1 = dead, 2 = vegetative state, 3 = lower severe disability, 4 = upper severe disability, 5 = lower moderate disability, 6 = upper moderate disability, 7 = lower good recovery, 8 = upper good recovery (recovery to pre-injury status).^22^

Six-month GOS-E was evaluated as the standard timepoint for outcomes in neurotrauma.^23^ Based on large recent TBI studies and neurosurgical clinical trials,^4^^,^^9^^,^^24^^,^^25^ GOS-E scores were dichotomised as unfavourable (GOS-E = 1–3) vs. favourable (GOS-E = 4–8). GOS-E = 4 was coded as “favourable” based on the premise that subjects with functional independence >8 h/day inside the home have considerable autonomy, and caregivers can maintain full-time employment and economic self-sufficiency.

### Statistical analysis

Categorical variables were reported using proportions. Continuous variables were assessed for normality, and reported as means and standard deviations when normally distributed (age, education years), and as medians and interquartile ranges (IQR) when non-normally distributed (Glasgow Coma Scale (GCS), Elixhauser Comorbidity Index, Rotterdam score, Injury Severity Score, length of stay, GOS-E). Differences between subgroups were assessed for statistical significance using chi-square test for categorical variables, analysis of variance for parametric continuous variables, and the Mann-Whitney U Test for non-parametric continuous variables. A two-sided threshold of p < 0.05 was used to define statistical significance. As this was a descriptive study, no adjustments were made for multiple comparisons. For sample size calculations, the available number of participants yielded a 95% confidence interval width on a percentage of no greater than: 12% for all surgery, 15% for decompressive craniectomy, and 25% for craniotomy. Statistical analyses were performed using SAS version 9.4 (SAS Institute, Cary, North Carolina, US).

### Role of the funding source

The funders of the study had no role in study design, data collection, data analysis, data interpretation, or writing of the report.

## Results

### Cranial surgery cohort characteristics

In 2032 subjects hospitalised with TBI from the TRACK-TBI Study, 260 (13%) underwent cranial surgery, comprising 65% decompressive craniectomy (N = 169), 23% craniotomy (N = 61), and 12% other surgeries (N = 30; skull base repair = 9, skull fracture repair = 9, intracranial pressure monitor placement in operating room = 6, other = 6). Mean age was 41.4 years (standard deviation 18, range of 17–89), 71% (1439) were male and 29% (593) were female. Demographic and clinical characteristics of subjects with and without cranial surgery are shown in Table 1. Subjects who underwent surgery presented with lower median emergency department GCS (6 [IQR 3–12] vs. 15 [IQR 14–15]), and higher proportions of severe TBI (61% (148/242) vs. 12% (214/1730)) and moderate TBI (15% (36/242) vs. 5% (84/1730)). Ten percent (155/1608) of subjects with two reactive pupils, 45% (14/31) with one unreactive pupil, and 57% (59/104) with two unreactive pupils underwent surgery. Ninety-nine percent (257/260) of the surgery cohort were admitted to ICU vs. 51% (904/1772) for non-surgery; the 3 subjects in the surgery cohort who were not admitted to ICU had skull fracture repair or debridement (not craniectomy/craniotomy). No statistically significant differences were observed in Elixhauser Comorbidity Index between surgery and non-surgery (p = 0.52).

**Table 1:** Demographic and clinical characteristics of the overall cohort, with and without cranial surgery.

Variable	Total (N = 2032)	Cranial surgery		
		No (N = 1772)	Yes (N = 260)	p-value
**Age (Years)**				
Mean (SD)	42.5 (18.0)	42.7 (18.2)	41.4 (16.6)	0.50
**Sex**				
Male	1439 (71%)	1231 (69%)	208 (80%)	0.0004
Female	593 (29%)	541 (31%)	52 (20%)	
**Race**				
Indian	5 (0%)	4 (0%)	1 (0%)	0.0009
Alaska Native/Inuit	1 (0%)	1 (0%)	0 (0%)	
Asian	70 (4%)	51 (3%)	19 (8%)	
Black	309 (15%)	286 (16%)	23 (9%)	
Hawaiian/Pacific Islander	6 (0%)	5 (0%)	1 (0%)	
White	1579 (79%)	1372 (79%)	207 (82%)	
Mixed race	28 (1%)	26 (1%)	2 (1%)	
Unknown	34	27	7	
**Ethnicity**				
Non-Hispanic	1560 (78%)	1362 (78%)	198 (78%)	0.95
Hispanic	437 (22%)	381 (22%)	56 (22%)	
Unknown	35	29	6	
**Education (Years)**				
Mean (SD)	13.1 (2.9)	13.2 (2.9)	12.6 (2.7)	0.014
Unknown	152	112	40	
**Employment status**				
Working now	1335 (70%)	1182 (71%)	153 (66%)	0.031
Disabled	58 (3%)	44 (3%)	14 (6%)	
Temporary leave	19 (1%)	17 (1%)	2 (1%)	
Keeping house	38 (2%)	35 (2%)	3 (1%)	
Unemployed	138 (7%)	112 (7%)	26 (11%)	
Student	88 (5%)	76 (5%)	12 (5%)	
Retired	206 (11%)	186 (11%)	20 (9%)	
Other	15 (1%)	14 (1%)	1 (0%)	
Unknown	135	106	29	
**Psychiatric history**				
No	1584 (78%)	1383 (78%)	201 (77%)	0.79
Yes	448 (22%)	389 (22%)	59 (23%)	
**Prior TBI history**				
No prior TBI	1458 (81%)	1279 (80%)	179 (84%)	0.38
Yes prior TBI—ED Visit	197 (11%)	178 (11%)	19 (9%)	
Yes prior TBI—Hospital admit	147 (8%)	133 (8%)	14 (7%)	
Unknown	230	182	48	
**Loss of consciousness**				
No	181 (9%)	169 (10%)	12 (5%)	0.011
Yes/Suspected	1735 (91%)	1506 (90%)	229 (95%)	
Unknown	116	97	19	
**Post-Traumatic amnesia**				
No	237 (14%)	222 (15%)	15 (8%)	0.020
Yes/Suspected	1474 (86%)	1307 (85%)	167 (92%)	
Unknown	321	243	78	
**ED arrival GCS**				
Median (IQR)	15 (13–15)	15 (14–15)	6 (3–12)	<0.0001
Severe TBI (GCS 3–8)	362 (18%)	214 (12%)	148 (61%)	<0.0001
Moderate TBI (GCS 9–12)	120 (6%)	84 (5%)	36 (15%)	
Mild TBI (GCS 13–15)	1490 (76%)	1432 (83%)	58 (24%)	
Unknown	60	42	18	
**ED arrival pupillary reactivity**				
Bilateral Reactive	1608 (92%)	1453 (96%)	155 (68%)	<0.0001
Unilateral unreactive	31 (2%)	17 (1%)	14 (6%)	
Bilateral unreactive	104 (6%)	45 (3%)	59 (26%)	
Unknown for either pupil	289	257	32	
**ED disposition**				
Hospital ward	871 (43%)	868 (49%)	3 (1%)	<0.0001
Intensive care unit	1161 (57%)	904 (51%)	257 (99%)	
**Extracranial ISS**				
Median (IQR)	4 (1–9)	4 (1–9)	4 (1–10)	0.57
Unknown	49	42	7	
**Elixhauser comorbidity index**				
Median (IQR)	1 (0–2)	1 (0–2)	1 (0–2)	0.52

Sociodemographic, medical history, and injury-related variables were compared between subjects hospitalised after acute TBI who underwent cranial surgery vs. those who did not. Column proportions (%) are shown. Variables with at least 5% missingness in the study sample were post-traumatic amnesia (16%), ED arrival pupillary reactivity (14%), prior TBI history (11%), education (7%), employment status (7%), and loss of consciousness (6%). ED, emergency department; GCS, Glasgow Coma Scale; IQR, interquartile range; ISS, Injury Severity Score; SD, standard deviation; TBI, traumatic brain injury; TRACK-TBI, Transforming Research and Clinical Knowledge in Traumatic Brain Injury.

Subjects with surgery had significantly higher proportions of all intracranial lesion types on CT (Table 2). Similarly, indicators of mass effect/herniation were elevated in the surgery cohort (midline shift ≥5 mm: 48% (115/238) vs. 2% (41/1688), cerebral oedema: 60% (142/238) vs. 8% (136/1687), cisternal effacement: 61% (145/238) vs. 4% (69/1688), as was the median Rotterdam Score (4 [IQR 3–5] vs. 2 [IQR 2–3]) (Table 2).

**Table 2:** Head CT characteristics of the overall cohort, with and without cranial surgery.

Variable	Total (N = 2032)	Cranial surgery		
		No (N = 1772)	Yes (N = 260)	p-value
**Epidural haematoma**				
No	1740 (90%)	1582 (94%)	158 (66%)	<0.0001
Yes	185 (10%)	105 (6%)	80 (34%)	
Unknown	107	85	22	
**Subdural haematoma**				
No	1287 (67%)	1244 (74%)	43 (18%)	<0.0001
Yes	638 (33%)	443 (26%)	195 (82%)	
Unknown	107	85	22	
**Subarachnoid haemorrhage**				
No	1103 (57%)	1066 (63%)	37 (16%)	<0.0001
Yes	823 (43%)	622 (37%)	201 (84%)	
Unknown	106	84	22	
**Cerebral contusion**				
No	1434 (74%)	1363 (81%)	71 (30%)	<0.0001
Yes	491 (26%)	324 (19%)	167 (70%)	
Unknown	107	85	22	
**Axonal injury**				
No	1713 (89%)	1519 (90%)	194 (82%)	0.0002
Yes	212 (11%)	168 (10%)	44 (18%)	
Unknown	107	85	22	
**Intraventricular haemorrhage**				
No	1807 (94%)	1596 (95%)	211 (89%)	0.001
Yes	119 (6%)	92 (5%)	27 (11%)	
Unknown	106	84	22	
**Midline shift**				
<5 mm	1770 (92%)	1647 (98%)	123 (52%)	<0.0001
≥5 mm	156 (8%)	41 (2%)	115 (48%)	
Unknown	106	84	22	
**Cerebral oedema**				
No	1647 (86%)	1551 (92%)	96 (40%)	<0.0001
Yes	278 (14%)	136 (8%)	142 (60%)	
Unknown	107	85	22	
**Cisternal effacement**				
None	1712 (89%)	1619 (96%)	93 (39%)	<0.0001
Partial effacement	143 (7%)	49 (3%)	94 (39%)	
Complete effacement	71 (4%)	20 (1%)	51 (21%)	
Unknown	106	84	22	
**Rotterdam CT score**				
Median (IQR)	2 (2–3)	2 (2–3)	4 (3–5)	<0.0001
1	40 (2%)	34 (2%)	6 (3%)	
2	1121 (58%)	1083 (64%)	38 (16%)	
3	568 (29%)	505 (30%)	63 (26%)	
4	86 (4%)	33 (2%)	53 (22%)	
5	67 (3%)	24 (1%)	43 (18%)	
6	44 (2%)	9 (1%)	35 (15%)	
Unknown	106	84	22	

Radiographic findings on initial head computed tomography (CT) scan were compared between subjects with TBI who underwent cranial surgery vs. those who did not. Column proportions (%) are shown. CT data were missing in 5% of the study sample. IQR, interquartile range; TBI, traumatic brain injury; TRACK-TBI, Transforming Research and Clinical Knowledge in Traumatic Brain Injury.

We then examined our target cohort who underwent cranial surgery for intracranial mass lesion and/or mass effect (craniectomy/craniotomy, N = 230).

### Decompressive craniectomy and craniotomy cohort: key comparisons in neuroimaging, hospitalisation, and outcomes

All subjects who underwent surgery for intracranial mass effect were admitted to ICU. Lesion types included subdural haematoma (86% (182/211)), subarachnoid haemorrhage (85% (180/211)), contusions (72% (151/211)), and epidural haematoma (35% (74/211)). For signs of mass effect/herniation, 54% (114/211) had midline shift ≥5 mm, 64% (134/211) had cerebral oedema, and 66% (140/211) had cisternal effacement. Sixty-seven percent (152/230) underwent intracranial pressure monitoring. Median ICU length of stay was 8 days and median hospital length of stay was 17 days (Table 3). In-hospital mortality was 20% (44/220), 25% (54/220) were discharged to home, 38% (76/220) to rehabilitation/skilled nursing facility, 10% (22/220) to long-term care, and 6% (13/220) to another hospital. Six-month outcomes included 29% (54/188) mortality, 2% (4/188) vegetative state, 23% (43/188) lower severe disability, 2% (3/188) upper severe disability, 29% (56/188) moderate disability, and 15% (28/188) good recovery (Table 3).

**Table 3:** Head CT, hospital and outcome characteristics of subjects with craniectomy/craniotomy.

Variable	Total (N = 230)	Cranial surgery type		
		DC (N = 169)	Craniotomy (N = 61)	p-value
**Epidural haematoma**				
No	137 (65%) [58, 71]	118 (75%) [67, 81]	19 (36%) [23, 50]	<0.0001
Yes	74 (35%) [29, 42]	40 (25%) [19, 33]	34 (64%) [50, 77]	
Unknown	19	11	8	
**Subdural haematoma**				
No	29 (14%) [9, 19]	12 (8%) [4, 13]	17 (32%) [20, 46]	<0.0001
Yes	182 (86%) [81, 91]	146 (92%) [87, 96]	36 (68%) [54, 80]	
Unknown	19	11	8	
**Subarachnoid haemorrhage**				
No	31 (15%) [10, 20]	17 (11%) [6, 17]	14 (26%) [15, 40]	0.005
Yes	180 (85%) [80, 90]	141 (89%) [83, 94]	39 (74%) [60, 85]	
Unknown	19	11	8	
**Cerebral contusion**				
No	60 (28%) [22, 35]	38 (24%) [18, 31]	22 (42%) [28, 56]	0.015
Yes	151 (72%) [65, 78]	120 (76%) [69, 82]	31 (58%) [44, 72]	
Unknown	19	11	8	
**Axonal injury**				
No	171 (81%) [75, 86]	127 (80%) [73, 86]	44 (83%) [70, 92]	0.67
Yes	40 (19%) [14, 25]	31 (20%) [14, 27]	9 (17%) [8, 30]	
Unknown	19	11	8	
**Intraventricular haemorrhage**				
No	97 (46%) [39, 53]	69 (44%) [36, 52]	28 (53%) [39, 67]	0.23
Yes	114 (54%) [47, 61]	89 (56%) [48, 64]	25 (47%) [33, 61]	
Unknown	19	11	8	
**Midline shift**				
<5 mm	97 (46%) [39, 53]	69 (44%) [36, 52]	28 (53%) [39, 67]	0.27
≥5 mm	114 (54%) [47, 61]	89 (56%) [48, 64]	25 (47%) [33, 61]	
Unknown	19	11	8	
**Cerebral oedema**				
No	77 (36%) [30, 43]	47 (30%) [23, 38]	30 (57%) [42, 70]	0.0009
Yes	134 (64%) [57, 70]	111 (70%) [62, 77]	23 (43%) [30, 58]	
Unknown	19	11	8	
**Cisternal effacement**				
None	71 (34%) [27, 40]	49 (31%) [24, 39]	22 (42%) [28, 56]	0.015
Partial effacement	89 (42%) [35, 49]	63 (40%) [32, 48]	26 (49%) [35, 63]	
Complete effacement	51 (24%) [19, 31]	46 (29%) [22, 37]	5 (9%) [3, 21]	
Unknown	19	11	8	
**Rotterdam CT score**				
Median (IQR)	4 (3–5)	4 (3–5)	3 (2–4)	<0.0001
1	6 (3%) [1, 6]	3 (2%) [0, 5]	3 (6%) [1, 16]	
2	29 (14%) [9, 19]	14 (9%) [5, 14]	15 (28%) [17, 42]	
3	47 (22%) [17, 28]	32 (20%) [14, 27]	15 (28%) [17, 42]	
4	52 (25%) [19, 31]	42 (27%) [20, 34]	10 (19%) [9, 32]	
5	42 (20%) [15, 26]	36 (23%) [16, 30]	6 (11%) [4, 23]	
6	35 (17%) [12, 22]	31 (20%) [14, 27]	4 (8%) [2, 18]	
Unknown	19	11	8	
**ED arrival GCS**				
Median (IQR)	6 (3–11)	6 (3–10)	8 (3–13)	0.07
A–Severe	134 (63%) [56, 69]	104 (66%) [58, 74]	30 (54%) [40, 67]	0.20
B–Moderate	34 (16%) [11, 22]	24 (15%) [10, 22]	10 (18%) [9, 30]	
C–Mild	45 (21%) [16, 27]	29 (18%) [13, 25]	16 (29%) [17, 42]	
Unknown	17	12	5	
**ED arrival pupillary reactivity**				
Bilateral reactive	135 (67%) [60, 74]	95 (64%) [55, 71]	40 (77%) [63, 87]	0.07
Unilateral unreactive	12 (6%) [3, 10]	9 (6%) [3, 11]	3 (6%) [1, 16]	
Bilateral unreactive	54 (27%) [21, 34]	45 (30%) [23, 38]	9 (17%) [8, 30]	
Unknown for either pupil	29	20	9	
**ICP monitor placement**				
Never	135 (67%) [60, 74]	95 (64%) [55, 71]	40 (77%) [63, 87]	0.0006
Before index surgery	12 (6%) [3, 10]	9 (6%) [3, 11]	3 (6%) [1, 16]	
During/After index surgery	54 (27%) [21, 34]	45 (30%) [23, 38]	9 (17%) [8, 30]	
**ICU LOS (Days)**				
Median (IQR)	8 (4–18)	13 (4–20)	4 (2–8)	0.0002
Unknown	20	18	2	
**Hospital LOS (Days)**				
Median (IQR)	17 (7–29)	20 (12–32)	8 (5–18)	<0.0001
Unknown	6	6	0	
**Discharge disposition**				
Home	54 (25%) [19, 31]	26 (16%) [11, 23]	28 (46%) [33, 59]	
Rehabilitation facility	73 (33%) [27, 40]	55 (35%) [27, 43]	18 (30%) [19, 43]	<0.0001
Skilled nursing facility	11 (5%) [3, 9]	8 (5%) [2, 10]	3 (5%) [1, 14]	
Nursing home	3 (1%) [0, 4]	3 (2%) [0, 5]	0 (0%) [0, 6]	
Long-Term acute care facility	22 (10%) [6, 15]	17 (11%) [6, 17]	5 (8%) [3, 18]	
Other hospital	13 (6%) [3, 10]	13 (8%) [4, 14]	0 (0%) [0, 6]	
Died	44 (20%) [15, 26]	37 (23%) [17, 31]	7 (11%) [5, 22]	
Unknown	10	10	0	
**GOS-E at six months**				
Median (IQR)	3 (1–6)	3 (1–5)	5 (3–7)	0.0001
1	54 (29%) [22, 36]	47 (33%) [26, 42]	7 (15%) [6, 28]	
2	4 (2%) [1, 5]	4 (3%) [1, 7]	0 (0%) [0, 8]	
3	43 (23%) [17, 30]	36 (26%) [19, 34]	7 (15%) [6, 28]	
4	3 (2%) [0, 5]	3 (2%) [0, 6]	0 (0%) [0, 8]	
5	29 (15%) [11, 21]	19 (13%) [8, 20]	10 (21%) [11, 36]	
6	27 (14%) [10, 20]	17 (12%) [7, 19]	10 (21%) [11, 36]	
7	13 (7%) [4, 12]	7 (5%) [2, 10]	6 (13%) [5, 26]	
8	15 (8%) [5, 13]	8 (6%) [2, 11]	7 (15%) [6, 28]	
Unknown	42	28	14	

Head computed tomography (CT) findings, length of stay (LOS), and outcome variables were compared between subjects with TBI who underwent cranial surgery for intracranial mass effect or mass lesion (DC/craniotomy). Column proportions (%) are shown. The 95% confidence intervals of the percentages are provided in brackets. Variables with at least 5% missingness were six-month GOSE (18%), ED pupils (13%), ICU LOS (9%), CT findings (8%), ED GCS (7%). DC = decompressive craniectomy; GOS-E, Glasgow Outcome Scale-Extended; ICP, intracranial pressure; ICU, intensive care unit; IQR, interquartile range; LOS, length of stay; TBI, traumatic brain injury.

**Fig. 2:**
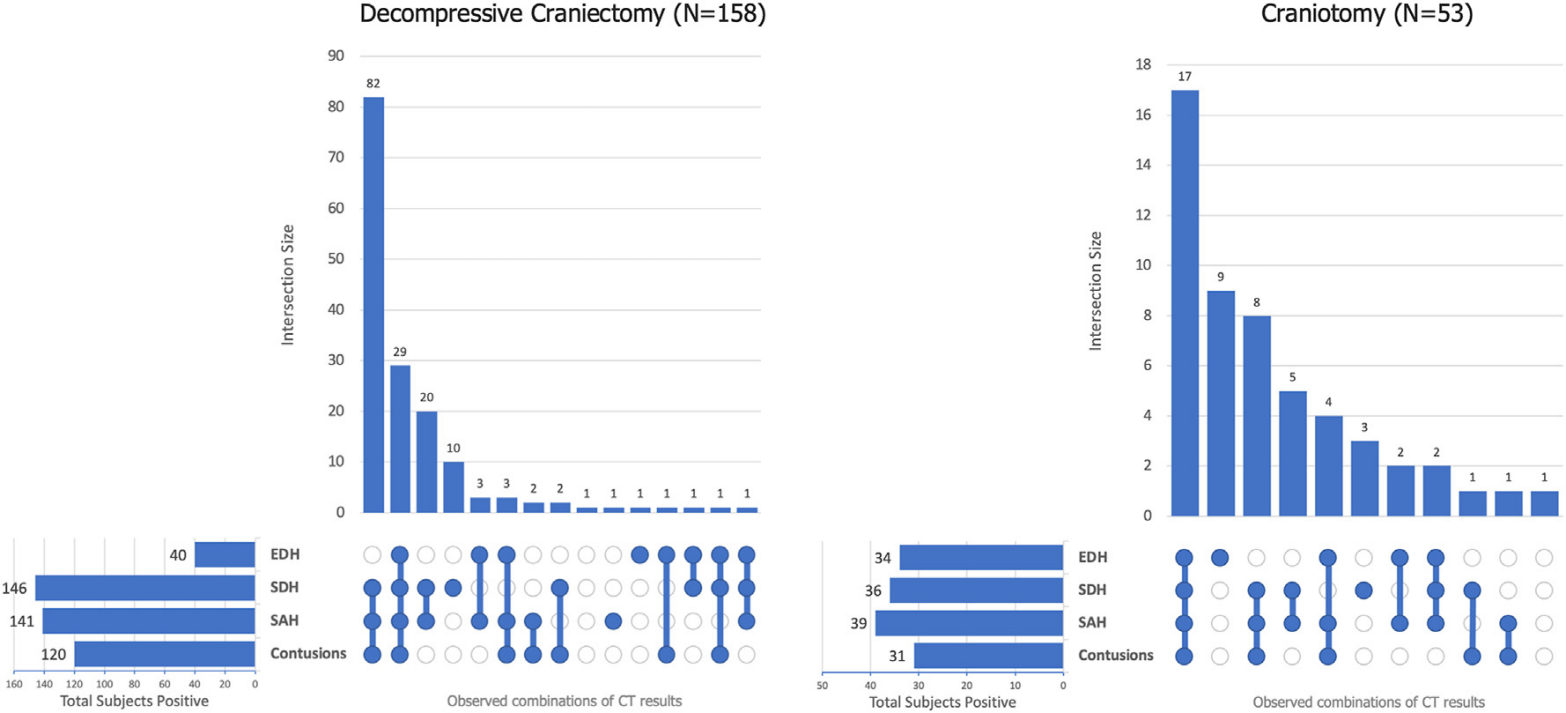
**Traumatic intracranial lesion types in the cranial surgery cohort**. UpSet plots were used to display concurrent traumatic intracranial lesion types on initial head CT scan in subjects who underwent decompressive craniectomy (DC) or craniotomy. Four primary TBI lesion types were examined (SDH, SAH, contusion, EDH). The sample size differs slightly from the full cohort (DC: N = 169, craniotomy: N = 61) as 11 subjects with DC and 8 subjects with craniotomy did not have complete data for CT lesion types and were not included in these plots. CT, computed tomography; EDH, epidural haematoma; SAH, subarachnoid haemorrhage; SDH, subdural haematoma; TRACK-TBI, Transforming Research and Clinical Knowledge in Traumatic Brain Injury.

Greater proportions of subdural haematoma, subarachnoid haemorrhage, and contusions were observed in decompressive craniectomy compared to craniotomy (92% (146/158) vs. 68% (36/53), p < 0.0001; 89% (141/158) vs. 74% (39/53), p = 0.015; 76% (120/158) vs. 58% (31/53), p = 0.015, respectively), while lower proportions of epidural haematoma were observed in decompressive craniectomy (25% (40/158) vs. 64% (34/53), p < 0.0001). For signs of mass effect/herniation, higher proportions of cerebral oedema and complete cisternal effacement were observed for decompressive craniectomy (70% (111/158) vs. 43% (23/53), p = 0.00085; 29% (46/158) vs. 9% (5/53), p = 0.015, respectively). Rotterdam Score and intracranial pressure monitoring rates were higher in decompressive craniectomy (median 4 vs. 3, p < 0.0001; 73% (124/169) vs. 46% (28/61), p = 0.0006, respectively) (Table 3). Concurrent lesion types by surgery type are shown in Fig. 2. The majority of injuries were consistent with multifocal TBI (≥2 lesion types: 92% (145/158) vs. 77% (41/53); ≥3 lesion types: 73% (116/158) vs. 58% (31/53), for decompressive craniectomy vs. craniotomy, respectively).

Median ICU length of stay (13 days [IQR 4–20] vs. 4 days [IQR 2–8], p = 0.0002) and hospital length of stay (20 days [IQR 12–32] vs. 8 days [IQR 5–18], p < 0.0001) were considerably longer in decompressive craniectomy compared to craniotomy. In-hospital mortality was significantly higher (23% (37/159) vs. 11% (7/61)) and discharge to home was significantly lower (16% (26/159) vs. 46% (28/61) for decompressive craniectomy compared to craniotomy. Median six-month GOS-E was significantly lower in decompressive craniectomy compared to craniotomy (3 [IQR 1–5] vs. 5 [IQR 3–7]), with higher proportions of unfavourable outcome (62% (87/141) vs. 30% (14/47), p < 0.0001) (Table 3).

### Decompressive Craniectomy and Craniotomy Cohort: associations with surgical timing

Overall, median time from injury to craniectomy/craniotomy was 1.8 h [IQR 1.1–5.0]; median time-to-decompressive craniectomy was 2.0 h [1.1–5.8] and to craniotomy was 1.5 h [1.0–3.3]. Most subjects with surgery underwent decompressive craniectomy or craniotomy within 4 h (69% (115/167) and 79% (48/61), respectively) and nearly all within 24 h (87% (146/167) and 97% (59/61), respectively). Age, comorbidities, extracranial Injury Severity Score, GCS, Rotterdam Score, intracranial pressure monitoring, length of stay, in-hospital mortality, and six-month GOS-E distributions by time-to-surgery are shown in Table 4.

**Table 4:** Clinical, hospital, and outcome characteristics of subjects with craniectomy/craniotomy, by surgery timing.

Variable	Timing of index cranial surgery since injury							
	0–2 Hours		2–4 Hours		4–24 Hours		>24 Hours	
	DC (N = 82)	Craniotomy (N = 36)	DC (N = 33)	Craniotomy (N = 12)	DC (N = 31)	Craniotomy (N = 11)	DC (N = 21)	Craniotomy (N = 2)
**Age (Years)**								
Mean (SD)	42.4 (16.5)	40.7 (16.1)	40.7 (17.9)	42.3 (15.9)	42.6 (16.4)	38.6 (16.6)	34.7 (14.0)	83.0 (7.8)
≥65 Years	8 (10%)	5 (14%)	4 (12%)	1	2 (6%)	0	0 (0%)	2
**ED arrival GCS**								
Median (IQR)	4 (3–8)	6 (3–9)	8 (3–14)	14 (12.5–15)	9 (3.25–13)	9 (6.5–13)	3 (3–6.5)	9 (6–12)
Unknown	5	4	4	1	1	0	1	0
**Rotterdam CT score**								
Median (IQR)	5 (4–6)	3 (2.25–4)	4 (2–4.5)	2 (2–3.5)	3 (3–5)	3 (2.25–3)	3 (3–4)	3 (2.75–4.25)
Unknown	7	6	2	1	2	1	0	0
**Extracranial ISS**								
Median (IQR)	1 (1–9)	1 (1–11)	5 (1–9.25)	1.5 (1–5)	5 (1–12.25)	1 (1–5.5)	5 (1–10)	5 (3–7)
**Elixhauser comorbidity index**								
Median (IQR)	1 (0–2)	0 (0–2)	1 (0–1)	0.5 (0–3.25)	1 (0–2)	0 (0–1)	1 (0–1)	2 (2–2)
**ICP Monitor**	59 (72%)	18 (50%)	20 (61%)	3	23 (74%)	6	20 (95%)	1
**Additional DC/Craniotomy**	6 (7%)	5 (14%)	4 (12%)	1	2 (6%)	1	2 (10%)	0
**Hospital LOS**								
Median (IQR)	17.8 (9.6–29.7)	9.3 (5.5–17.3)	13.9 (6.8–22.3)	5.4 (3.3–6.6)	27.3 (13.5–55.2)	14.5 (4.9–21.3)	34.3 (19.2–46.5)	23.9
**In-Hospital mortality**	22 (28%)	5 (14%)	6 (18%)	1	5 (17%)	0	4 (19%)	1
**GOS-E at six months**								
Median (IQR)	3 (1–5)	5 (3–6)	3 (1.5–6)	6 (5–6)	3 (2–5)	6.5 (5–7.75)	3 (1–3)	2 (1.5–2.5)
Unfavourable (GOS-E 1–3)	44 (63%)	8 (32%)	14 (52%)	2	13 (54%)	2	14 (78%)	2
Favourable (GOS-E 4–8)	26 (37%)	17 (68%)	13 (48%)	8	11 (46%)	8	4 (22%)	0
Unknown	12	11	6	2	7	1	3	0

Age, clinical factors, neurologic and extracranial injury severity, LOS, in-hospital mortality, and six-month outcome variables are shown descriptively for subjects with TBI who underwent cranial surgery for intracranial mass effect or mass lesion (DC/craniotomy) across time-to-surgery subgroups (0–2, 2–4, 4–24, and >24 h since injury). Two subjects had unknown timing and were not included in this table. Percentages were not calculated for variable categories with denominator <20. DC, decompressive craniectomy; ED, emergency department; GCS, Glasgow Coma Scale; GOS-E, Glasgow Outcome Scale-Extended; ICP, intracranial pressure; IQR, interquartile range; ISS, Injury Severity Score; LOS, length of stay; SD, standard deviation; TBI, traumatic brain injury.

After the index craniectomy or craniotomy, 9% (21/228) required additional craniectomy or craniotomy to relieve mass effect (8% (14/167) and 11% (7/61), respectively). For subjects with an index craniotomy (7 of 21; 4 for subdural haematoma, 2 for epidural haematoma, 1 for contusion), the second surgery comprised 5 ipsilateral craniectomies, 1 ipsilateral craniotomy for re-evacuation of haemorrhage, and 1 contralateral craniectomy; in this subgroup, 86% of second surgeries were ipsilateral and 14% were contralateral to the index surgery. For subjects with an index decompressive craniectomy (14 of 21), 6 underwent additional ipsilateral decompression, 4 contralateral craniectomy, 3 contralateral craniotomy, and 1 unspecified craniotomy; in subjects with known laterality for the second surgery, 46% were ipsilateral and 54% were contralateral to their index surgery. The distribution of ipsilateral vs. contralateral second surgery did not statistically significantly differ between index decompressive craniectomy and index craniotomy (p = 0.16). Hospital mortality (20% (4/20) vs. 20% (40/205), p = 0.99), six-month mortality (21% (4/19) vs. 30% (50/167), p = 0.60), and six-month unfavourable outcome (78% (15/19) vs. 59% (99/167), p = 0.14) did not statistically significantly differ for those with vs. without a second cranial surgery, respectively.

Within the first 24 h post-injury, earlier decompressive craniectomy was associated with greater injury burden (median; 0–2 h: GCS = 4, Rotterdam Score = 5; 2–4 h: GCS = 8, Rotterdam Score = 4; 4–24 h: GCS = 9, Rotterdam Score = 3); the same trend was not observed for craniotomy (median; 0–2 h: GCS = 6, Rotterdam Score = 3; 2–4 h: GCS = 14, Rotterdam Score = 2; 4–24 h: GCS = 9, Rotterdam Score = 3) (Table 4). Intracranial pressure monitoring rates were comparable across subgroups within 0–24 h. Extracranial Injury Severity Score was higher in subjects who underwent decompressive craniectomy at 2–24 h vs. 0–2 h (median [IQR]: 5.0 [1.0–10.8] vs. 1.0 [1.0–9.0], p = 0.049) (Table 4). In-hospital mortality (0–2 h: 27% (22/82) vs. 2–24 h: 17% (11/64) and six-month unfavourable outcome (0–2 h: 63% (44/82) vs. 2–24 h: 53% (27/51)) were highest for subjects undergoing decompressive craniectomy within 0–2 h compared to later time intervals, and were approximately twice as high in decompressive craniectomy compared to craniotomy. Rates of mortality and unfavourable outcome were comparable between 2–4 and 4–24 h, and similarly higher in decompressive craniectomy vs. craniotomy. Hospital length of stay was generally at least twice as long for subjects with decompressive craniectomy vs. craniotomy (Table 4).

## Discussion

Our study describes the clinical profile and outcomes of an 18-centre cohort of patients hospitalised with acute TBI who underwent cranial surgery from the TRACK-TBI Study, which utilised the strengths of prospective enrolment and rigorous data standardisation conforming to the NINDS TBI Common Data Elements.^16^ The rate of 13% with cranial surgery and 11% with decompressive craniectomy or craniotomy in our cohort of 2032 patients is comparable to historical reports.^26^ Not unexpectedly, patients who required cranial surgery were more likely to present with 1) greater clinical injury by GCS classification, 2) all types of traumatic intracranial lesions on CT, and 3) increased radiologic burden of intracranial injury and mass effect. Most patients who underwent surgery underwent decompressive craniectomy (65%), and 72% underwent surgery within 4 h. Higher clinical and radiologic TBI severity was associated with earlier time-to-surgery in patients who underwent decompressive craniectomy. Compared to craniotomy, decompressive craniectomy was associated with increased ICU and hospital length of stay and six-month unfavourable outcome, likely reflective of intracranial injury severity. These findings provide an overview of the contemporary surgical practice for TBI across US Level I trauma centres and contribute to the evidence base for future hypothesis-driven investigations in risk stratification, prognostication, and comparative effectiveness research.

### Clinical and radiologic characteristics

Within the TRACK-TBI cohort, there were no significant differences between the surgical vs. non-surgical cohorts for age, Elixhauser Comorbidity Index, psychiatric history, and incidence of prior TBI. Not surprisingly, patients who underwent cranial surgery were more likely to be male, have severe TBI on clinical and radiologic assessments, and require ICU admission. Male sex has been associated with risky behaviour and increased risk of trauma.^27^ Forty-five percent of subjects with unilateral unreactive pupil and 57% with bilateral unreactive pupils upon arrival to the emergency department underwent cranial surgery. The TRACK-TBI Study did not have data on changes in pupillary reactivity after treatment (e.g. hyperosmolar therapy, surgery) which is a limitation. Nevertheless, our finding supports in-depth characterisation of pupillary reactivity and its changes to better understand responsiveness to therapy, surgical decision-making, and outcomes, especially in patients with bilateral unreactive pupils (a poor prognostic sign).^6^^,^^7^ A lower proportion of patients who underwent cranial surgery were Black (9% vs. 16%). Reasons for this are not well understood and may relate to US TBI casemix and medical decision-making in the emergency setting, which require investigation.

The neurosurgical practice across the 18 TRACK-TBI centres generally conformed to the Brain Trauma Foundation Guidelines for surgical treatment of brain compression and herniation.^5^ Patients who underwent surgery were more likely to present with traumatic subarachnoid haemorrhage, subdural haematoma, and parenchymal contusions, corroborating literature on the co-occurrence of these lesion types and their deleterious associations with outcome.^28^ Surgical patients had higher frequencies of axonal injury and intraventricular haemorrhage, which are caused by rotational and/or greater traumatic forces to the brain and predict poorer outcomes.^29^ Markers of brain compression e.g. midline shift >5 mm and cisternal effacement were elevated in our surgical cohort.

Epidural haematomas were more frequent in the surgical cohort and notably presented predominantly with multifocal TBI. As shown in Fig. 2, only 14% (10/74) of epidural haematomas were isolated, i.e. did not co-occur with subdural haematoma, subarachnoid haemorrhage, or contusion. Our cohort had a slightly higher proportion of epidural haematomas compared to prior reports citing an 8% prevalence in TBI.^30^^,^^31^ Our results showed benefits of surgical evacuation for primarily isolated epidural haematomas compared to prior reports.^32^ Changes in the current TBI casemix compared with prior cohorts from 15 to 40 years ago, in conjunction with improved sensitivity of modern CT scanners and earlier injury detection due to efficacy of prehospital transport to US Level I trauma centres, may have contributed to the increased prevalence of epidural haematomas in TRACK-TBI and of non-isolated epidural haematomas in the surgical cohort. Conversely, a greater proportion of epidural haematomas in our study were treated with craniotomies than decompressive craniectomies (Table 3, 64% vs. 25%), consistent with current standard of care practices for bone flap replacement after uncomplicated haematoma evacuations whereas craniectomies are more common in multifocal injuries with parenchymal swelling, which often include subdural haematomas and contusions (Fig. 2). Our understanding of the landscape of epidural haematomas is changing. In a 2024 CENTER-TBI study of 461 patients with TBI with epidural haematomas across 65 European hospitals, 71% had concurrent subdural haematomas and/or contusions, showing that epidural haematomas can no longer be presumed as mostly isolated.^33^ Taken together with the RESCUE-ASDH data on craniectomy vs. craniotomy in cases of clinical equipoise,^34^ emerging evidence from recent large multicentre studies emphasises the importance of re-examining historical and new factors that may improve guidance for surgical decision-making.

### Surgery type, timing, and outcomes

Our study focused on the two primary surgery types for patients with TBI and intracranial mass effect: decompressive craniectomy and craniotomy. Patients who underwent decompressive craniectomy had higher intracranial injury severity, indicating more urgent need for decompression. Accordingly, 73% (116/158) of subjects with decompressive craniectomy and 58% (31/53) with craniotomy had ≥3 major lesion types in our cohort, showing that current surgical indications inherently comprise many patients with multifocal TBI rather than isolated lesions. As shown in Table 3, the decompressive craniectomy group had considerably higher proportions with discrete injury types (subdural haematoma, subarachnoid haemorrhage, contusions; 76–92% vs. 58–74%), mass effect/herniation (complete cisternal effacement: 29% vs. 9%; cerebral oedema: 70% vs. 43%), overall intracranial injury severity (Rotterdam score: median 4 vs. 3), and intracranial pressure monitoring (73% vs. 46%), compared to the craniotomy group. These data clearly support the conventional wisdom that decompressive craniectomies are reserved for cases with herniation and refractory intracranial mass effect, while craniotomies are utilised when intracranial mass effect originates from an evacuable lesion. Our US study corroborates recent high-quality European studies on surgical indication and comparative effectiveness of decompressive craniectomy and craniotomy in subdural haematoma,^4^^,^^11^ and provides the rationale to extend these investigations to other traumatic lesions.

Our study provides several important findings regarding time-to-surgery. Overall median time-to-surgery was <2 h, the majority (71%) underwent surgery within 4 h, and nearly all (90%) within 24 h, supporting the notion that US Level I trauma centres with neurosurgery possess the resources and expertise to expeditiously take patients with neurological decompensation to surgery. Within the first 4 h, those with faster time-to-surgery (0–2 h) were more neurologically injured, i.e. had considerably lower GCS (median; decompressive craniectomy: 4 vs. 8; craniotomy: 6 vs. 14), higher Rotterdam Scores (decompressive craniectomy: 5 vs. 4; craniotomy: 3 vs. 2), and higher rates of intracranial pressure monitoring (decompressive craniectomy: 72% vs. 61%, craniotomy: 50% vs. 25%). Findings from our US cohort suggest that patients undergo prioritisation for early surgical decompression and intracranial pressure monitoring based on neurological injury severity, even at hyper-acute timeframes of 0–4 h. Interestingly, the relationship between injury severity and time-to-surgery is less clear for 4–24 h: patients with decompressive craniectomy in this subgroup had higher GCS and lower Rotterdam Scores than the 0–2 h and 2–4 h subgroups but also the highest (74%) intracranial pressure monitoring rates, while patients who underwent craniotomy between 4 and 24 h are about as injured as those who underwent craniotomy between 0 and 2 h. Notably, some patients with surgery did not undergo intracranial pressure monitoring, which may occur in cases where the postoperative neurological exam can be followed conservatively, or adequate evacuation of a mass lesion with little concern for elevated intracranial pressure after surgery. Potential explanations for delays in cranial surgery include the need for clinical stabilisation prior to neurosurgical intervention, intracranial pressure elevation not immediately refractory to medical management, and delayed clinical or radiographic neuroworsening,^35^ which warrant further study. Median extracranial Injury Severity Scores were marginally higher in subjects with decompressive craniectomy vs. craniotomy at 2–24 h (5 vs. 1), and those with decompressive craniectomy at 2–24 h vs. 0–2 h (5 vs. 1). It is plausible that patients with intracranial injury requiring decompressive craniectomy and concomitant greater than minimal extracranial injury severity underwent urgent evaluation and stabilisation for multisystem trauma prior to decompressive craniectomy. Efficacies of expedient and well-resourced emergency trauma care are regularly benchmarked across Level I trauma centres.^36^

In considering outcomes for cranial surgery within 24 h, in-hospital mortality and six-month unfavourable outcome are highest for 0–2 h, and twice as high in decompressive craniectomy vs. craniotomy. Rates of mortality and unfavourable outcome were comparable between 2–4 and 4–24 h, and higher for decompressive craniectomy compared to craniotomy. Hospital length of stay was generally two-fold greater for decompressive craniectomy compared to craniotomy. As stated previously, the distribution of these outcomes underscores the relationships between intracranial injury severity, surgical timing, and need for decompressive craniectomy. The 21 patients who underwent decompressive craniectomy after 24 h had the lowest presenting GCS (median = 3) of all subgroups despite having comparable presenting injury severity with other subgroups (median Rotterdam Score = 3, median extracranial Injury Severity Score = 5). Mortality for the >24 h subgroup was comparable to 4–24 h, while the median hospital length of stay (34 days) and rate of unfavourable outcomes were the highest amongst time-to-surgery subgroups (78%). These results indicate that patients in this subgroup require closer evaluation, as they may have confounding reasons for delayed surgery (e.g. need for systemic stabilisation, interval neuroworsening)^37^ that confer poorer outcomes without significant differences in mortality.

Recent data have provided actionable updates to historical neurosurgical practices.^4^^,^^13^^,^^14^^,^^34^^,^^38^, ^39^, ^40^ The 2023 RESCUE-ASDH trial suggested that in cases without extensive intraoperative brain swelling, bone flap replacement can be considered during the index surgery for acute subdural haematoma, with comparable 6- and 12-month functional outcomes in decompressive craniectomy and craniotomy groups.^34^ However, additional cranial surgery was performed in 15% vs. 7%, while wound complications occurred in 4% vs. 12%, of index craniotomy vs. craniectomy cases, respectively.^4^ Our smaller surgical cohort showed an overall 9% reoperation rate, which did not statistically significantly differ by index surgery type or laterality. Decision-making for primary and secondary cranial surgeries are contingent on the specific presentation and evolution of each patient case and challenging to ascertain from research data. Future research in surgical neurotrauma would benefit from inclusion of standardised data fields for intraoperative observations regarding clinical equipoise (e.g. whether brain swelling beyond the limits of the bone flap defect was or was not observed) and the specific causal events of return to operating room (e.g. bone flap removal due to residual mass effect).

### Prioritising next steps in TBI clinical research

Our study sets the stage for future investigations into the clinical and imaging factors influencing surgical management and outcomes. Characterisation of demographic factors, such as younger vs. older age,^39^, ^40^, ^41^, ^42^ lesion type, location and severity in decompressive craniectomy and craniotomy may provide insight into optimising surgical decision-making pathways for acute TBI, such as surgery type and timing and their relationship to outcomes.^43^ In cranial surgery for multifocal TBI, coding the primary surgical indication will aid the ability to subgroup patients by the primary lesion type(s), and clarify surgical decision-making – these codes were not systematically captured in TRACK-TBI, and represents an important area for refinement within the TBI Common Data Elements^16^ and other ongoing data standardisation efforts in neurotrauma research and clinical care.^44^ Factors that may differentially affect time-to-surgery in craniectomy vs. craniotomy, e.g. initial resuscitation, intracranial pressure monitor placement, extracranial surgery types/timing (e.g. complex polytrauma), evolution of secondary injury, and frailty, will be important to delineate whether a patient is indicated for surgical intervention. Efforts towards refining TBI prognostication models with contemporary data and updated clinical measures are needed to advance surgical management of TBI in emergency department and hospital settings. Decision-making appears to be more complex for patients in the 4–24 h and >24 h subgroups in our study, and these are precisely the cohorts requiring deeper examination in hypothesis-driven studies to determine the predictors that may encourage clinicians to elect surgical or medical management. Importantly, evaluating associations between diagnostic clinical and laboratory factors, e.g. blood-based brain injury biomarkers with robust associations with radiographic intracranial injury severity and prognosis,^4^^,^^11^^,^^13^^,^^28^ may aid in the refinement of prognostic models and influence treatment recommendations in patients with TBI who require cranial surgery.

### Limitations

We recognise several limitations of our study. The TRACK-TBI Study enrolled subjects with TBI through convenience sampling, which inherently limits generalisability. Our data collected from a large cohort of patients with acute TBI presenting to US Level I trauma centres may not be representative of other medical centres or populations, such as those in rural communities or non-US countries. The Global Neurotrauma Outcomes Study, conducted across 159 hospitals in 57 countries between 2018 and 2020, reported considerable variability in characteristics and management in TBI patients requiring cranial surgery.^45^ Similarly, our data adds to the evidence regarding the complexity of surgical characteristics and outcomes for patients with TBI in the Americas. In our study, surgical decision-making was determined at the discretion of the managing surgeon, which is inherently subject to selection bias. While certain patients present with clear surgical indications and should not be influenced by selection biases (e.g. GCS 3–8 with multifocal TBI, cerebral oedema and herniation requiring hemicraniectomy), other cases require nuanced decision-making and have several treatment options (e.g. GCS 14 with 8 mm subdural haematoma who could undergo evacuation by burr hole, craniotomy, craniectomy, or bedside craniostomy at the surgeon's discretion and/or institutional resource availability). Similarly, while current TBI management guidelines are based on the best available evidence (e.g. Brain Trauma Foundation Guidelines, Seattle International Severe Traumatic Brain Injury Consensus Conference),^5^^,^^35^ and expert consensus, there remains locoregional practice variations which may influence clinical decision-making and data collection.^46^ We acknowledge that our results, including indications for surgery, surgery type/timing, and intracranial pressure monitoring, were inherently biased and limited by best practices and/or institutional standards for TBI care. Our primary aim was to provide a descriptive overview of a representative modern US surgical TBI sample, and to establish the evidentiary groundwork for hypothesis-driven research of targeted questions in specific TBI cohorts. Therefore, multivariable regression models and estimates of risk were not pursued, and relationships between reported factors and potential underlying confounders were not accounted for. We recognise that several variables with importance in TBI prognostication, such as lesion location and volume, cranial and extracranial injury progression, CT-occult MRI features, brain injury-specific blood-based biomarker levels, and additional responses to treatment were not assessed in our descriptive study.^1^^,^^47^ In particular, discrete intracranial injury types may confer differing severities and consequences depending on location and injury pattern—a critical area for near-term investigations with quantitative neuroimaging data. Extracranial surgery types/timing and need for massive transfusion in critical trauma^48^ were not evaluated and warrant examination. We also recognise there were varying levels of missingness in some of our descriptive variables, which raises the risk of selection bias inherent in our study sample. TBI casemix, clinical, and socioeconomic resources may vary significantly by country or locale and impact institutional care quality.^45^ Outcomes may change after cranioplasty,^49^ which has variable timing due to institutional and provider practices, was not routinely queried during TRACK-TBI follow-up, and should be considered in future trial methodologies analysing decompressive craniectomy. It will be important to establish adequately powered sample sizes *a priori* to answer specific hypothesis-driven questions in future research.

### Conclusions

In a prospective, 18-centre cohort of patients who presented to US trauma centres and were hospitalised for TBI, the incidence of overall cranial surgery and surgical decompression/evacuation were 13% and 11%, respectively, comparable to prior reports. Surgical decision-making, including timing (0–2, 2–4, 4–24, >24 h) and type of decompression/evacuation, generally aligned with intracranial injury severity. Multifocal TBIs were predominant. Most patients (71%) underwent surgery for mass effect within 4-h of injury and underwent intracranial pressure monitoring (66%). These findings summarise the contemporary TBI surgical practice across US trauma centres and provide the foundation for hypothesis-driven analyses in targeted subpopulations.

## Contributors

Conceptualisation: JKY, JHK, MCH, TAVE, NRT, GTM; Data Curation: JKY, JHK, JKB, TAVE, NRT, GTM; Formal Analysis: JKY, JHK, JKB, MCH, TAVE, MME, NRT, GTM; Funding Acquisition: JKY, GTM; Investigation: JKY, JHK, JKB, MCH, TAVE, MME, NRT, GTM; Methodology: JKY, JKB, NRT, GTM; Project Administration: JKY, JKB, NRT, GTM; Resources: JKY, JKB, NRT, GTM; Software: JKY, JKB, NRT, GTM; Supervision: JKY, JHK, JKB, MCH, TAVE, DOO, NRT, GTM; Validation: JKY, JHK, JKB, MCH, TAVE, MME, BF, PJB, DP, XS, AMP, SJ, DOO, NRT, GTM; Visualisation: JKY, JKB; Writing–Original Draft: JKY, JHK, JKB, MCH, TAVE, MME, BF, FKK, PJB, DP, YML, RSK, MJV, XS, GGS, JCW, ARF, JRH, KKWW, HD, VYW, YGB, SRT, DYM, MAM, LBN, AMD, PET, MBS, AMP, JTG, RDA, HFL, PM, ELY, CSR, DKM, AIRM, AJM, SJ, DOO, NRT, GTM; Writing–Review and Editing: JKY, JHK, JKB, MCH, TAVE, MME, BF, FKK, PJB, DP, YML, RSK, MJV, XS, GGS, JCW, ARF, JRH, KKWW, HD, VYW, YGB, SRT, DYM, MAM, LBN, AMD, PET, MBS, AMP, JTG, RDA, HFL, PM, ELY, CSR, DKM, AIRM, AJM, SJ, DOO, NRT, GTM, TRACK-TBI Investigators Author Block.

Authors who have directly accessed and verified the underlying data reported in the manuscript: JKY, JHK, JKB, NRT, GTM. All authors (JKY, JHK, JKB, MCH, TAVE, MME, BF, FKK, PJB, DP, YML, RSK, MJV, XS, GGS, JCW, ARF, JRH, KKWW, HD, VYW, YGB, SRT, DYM, MAM, LBN, AMD, PET, MBS, AMP, JTG, RDA, HFL, PM, ELY, CSR, DKM, AIRM, AJM, SJ, DOO, NRT, GTM, TRACK-TBI Investigators Author Block) confirm that they had full access to all the data in the study. All authors accept responsibility for submission of the manuscript for publication.

## TRACK-TBI investigators author block

**(Study Group, to be indexed as “Collaborators”):** Jason E. Chung, MD, PhD (Department of Neurological Surgery, University of California, San Francisco, San Francisco, California, United States); Bukre Coskun, BA (Department of Neurological Surgery, University of California, San Francisco, San Francisco, California, United States); Shawn R. Eagle, PhD (Department of Neurological Surgery, University of Pittsburgh Medical Center, Pittsburgh, Pennsylvania, United States); Leila L. Etemad, BA (Department of Neurological Surgery, University of California, San Francisco, San Francisco, California, United States); Brian Fabian, MPA (Department of Neurological Surgery, University of California, San Francisco, San Francisco, California, United States); V. Ramana Feeser, MD (Department of Emergency Medicine, Virginia Commonwealth University, Richmond, Virginia, United States); Shankar Gopinath, MD (Department of Neurological Surgery, Baylor College of Medicine, Houston, Texas, United States); Christine J. Gotthardt, MS (Department of Neurological Surgery, University of California, San Francisco, San Francisco, California, United States); Ramesh Grandhi, MD, MS (Department of Neurological Surgery, University of Utah Medical Center; Salt Lake City, Utah, United States); Sabah Hamidi, BA (Department of Neurological Surgery, University of California, San Francisco, San Francisco, California, United States); Ruchira M. Jha, MD, MSc (Department of Neurology, Barrow Neurological Institute, Phoenix, Arizona, United States); Christopher Madden, MD (Department of Neurological Surgery, University of Texas Southwestern Medical Center, Dallas, Texas, United States); Randall Merchant, PhD (Department of Anatomy, Virginia Commonwealth University, Richmond, Virginia, United States); Lindsay D. Nelson, PhD (Department of Neurological Surgery, Medical College of Wisconsin, Milwaukee, Wisconsin, United States); Richard B. Rodgers, MD (Goodman Campbell Brain and Spine, Carmel, Indiana, United States); Andrea L. C. Schneider, MD, PhD (Department of Neurology, University of Pennsylvania, Philadelphia, Pennsylvania, United States); David M. Schnyer, PhD (Department of Psychology, University of Texas at Austin, Austin, Texas, United States); Abel Torres-Espin, PhD (Department of Neurological Surgery, University of California, San Francisco, San Francisco, California, United States); Joye X. Tracey, BS (Department of Neurological Surgery, University of California, San Francisco, San Francisco, California, United States); Alex B. Valadka, MD (Department of Neurological Surgery, University of Texas Southwestern Medical Center, Dallas, Texas, United States); Ross D. Zafonte, DO (Department of Rehabilitation Medicine, Harvard Medical School, Boston, Massachusetts, United States).

## Data sharing statement

Data from the TRACK-TBI Study are available through the Federal Interagency Traumatic Brain Injury Research (FITBIR) Informatics System at https://doi.org/10.23718/FITBIR/1518881. Qualified researchers can request access to data stored in FITBIR, which requires obtaining data access privileges as outlined by FITBIR. TRACK-TBI study protocols and data collection forms are available at https://tracktbi.ucsf.edu/researchers. Investigators interested in the investigation of specific data elements may submit a Data Collaboration Request to the TRACK-TBI Executive Committee through the process outlined at https://tracktbi.ucsf.edu/collaboration-opportunities. Statistical analyses were supervised by Dr. Nancy R. Temkin, PhD, Professor of Neurological Surgery and Professor of Biostatistics at University of Washington (Seattle, Washington, United States). Analytic code used to conduct the analyses presented in this study are not available in a public repository and may be made available upon request by emailing the corresponding author. TRACK-TBI study protocols, informed consent forms, data collection forms, and data dictionaries are available for public access at: https://tracktbi.ucsf.edu/researchers.

## Declaration of interests

AMD declares: grant funding from the Mercatus Center at George Mason University (not related to the current work). DKM declares: grant funding from United Kingdom National Institute for Health Research (not related to the current work). KKWW declares: is a shareholder of Gryphon Bio, Inc. (not related to the current work). All other authors declare no conflict of interest.
